# Development of a Novel, Genome Subtraction-Derived, SARS-CoV-2-Specific COVID-19-nsp2 Real-Time RT-PCR Assay and Its Evaluation Using Clinical Specimens

**DOI:** 10.3390/ijms21072574

**Published:** 2020-04-08

**Authors:** Cyril Chik-Yan Yip, Chi-Chun Ho, Jasper Fuk-Woo Chan, Kelvin Kai-Wang To, Helen Shuk-Ying Chan, Sally Cheuk-Ying Wong, Kit-Hang Leung, Agnes Yim-Fong Fung, Anthony Chin-Ki Ng, Zijiao Zou, Anthony Raymond Tam, Tom Wai-Hin Chung, Kwok-Hung Chan, Ivan Fan-Ngai Hung, Vincent Chi-Chung Cheng, Owen Tak-Yin Tsang, Stephen Kwok Wing Tsui, Kwok-Yung Yuen

**Affiliations:** 1 Department of Microbiology, Queen Mary Hospital, HKSAR, Hong Kong, China; yipcyril@hku.hk (C.C.-Y.Y.); cwh366@ha.org.hk (T.W.-H.C.); vcccheng@hku.hk (V.C.-C.C.); 2 Genomics and Bioinformatics Programme, The Chinese University of Hong Kong, HKSAR, Hong Kong, China; hkhcc@link.cuhk.edu.hk; 3 State Key Laboratory of Emerging Infectious Diseases, The University of Hong Kong, HKSAR, Hong Kong, China; jfwchan@hku.hk (J.F.-W.C.); kelvinto@hku.hk (K.K.-W.T.); chankh2@hku.hk (K.-H.C.); 4 Department of Microbiology, The University of Hong Kong, HKSAR, Hong Kong, China; khl17@hku.hk (K.-H.L.); agnes_fung@hku.hk (A.Y.-F.F.); anthonyng912@gmail.com (A.C.-K.N.); jozou0929@gmail.com (Z.Z.); 5 Department of Clinical Microbiology and Infection, The University of Hong Kong-Shenzhen Hospital, Shenzhen 518053, China; 6 Carol Yu Centre for Infection, Li Ka Shing Faculty of Medicine, The University of Hong Kong, HKSAR, Hong Kong, China; 7 Department of Medicine, Queen Elizabeth Hospital, HKSAR, Hong Kong, China; csy249a@ha.org.hk; 8 Department of Pathology, Queen Elizabeth Hospital, HKSAR, Hong Kong, China; wcy288@ha.org.hk; 9 Department of Medicine, Queen Mary Hospital, HKSAR, Hong Kong, China; antamwf@connect.hku.hk; 10 Department of Medicine, The University of Hong Kong, HKSAR, Hong Kong, China; ivanhung@hku.hk; 11 Department of Medicine and Geriatrics, Princess Margaret Hospital, HKSAR, Hong Kong, China; tsangty@ha.org.hk; 12 School of Biomedical Sciences, The Chinese University of Hong Kong, HKSAR, Hong Kong, China; kwtsui@cuhk.edu.hk

**Keywords:** SARS-CoV-2, COVID-19, nsp2, real-time RT-PCR, genome subtraction, GolayMetaMiner, sensitivity, specificity, clinical evaluation, COVID-19-nsp2 assay

## Abstract

The pandemic novel coronavirus infection, Coronavirus Disease 2019 (COVID-19), has affected at least 190 countries or territories, with 465,915 confirmed cases and 21,031 deaths. In a containment-based strategy, rapid, sensitive and specific testing is important in epidemiological control and clinical management. Using 96 SARS-CoV-2 and 104 non-SARS-CoV-2 coronavirus genomes and our in-house program, GolayMetaMiner, four specific regions longer than 50 nucleotides in the SARS-CoV-2 genome were identified. Primers were designed to target the longest and previously untargeted nsp2 region and optimized as a probe-free real-time reverse transcription-polymerase chain reaction (RT-PCR) assay. The new COVID-19-nsp2 assay had a limit of detection (LOD) of 1.8 TCID_50_/mL and did not amplify other human-pathogenic coronaviruses and respiratory viruses. Assay reproducibility in terms of cycle threshold (Cp) values was satisfactory, with the total imprecision (% CV) values well below 5%. Evaluation of the new assay using 59 clinical specimens from 14 confirmed cases showed 100% concordance with our previously developed COVID-19-RdRp/Hel reference assay. A rapid, sensitive, SARS-CoV-2-specific real-time RT-PCR assay, COVID-19-nsp2, was developed.

## 1. Introduction

Coronaviruses are positive sense, single-stranded RNA viruses that cause important diseases in human and animals [1]. In the past two decades, at least three novel human-pathogenic coronaviruses have crossed species barriers to cause major epidemics. These included severe acute respiratory syndrome coronavirus (SARS-CoV), Middle East respiratory syndrome coronavirus (MERS-CoV), and the most recently emerged severe acute respiratory syndrome coronavirus 2 (SARS-CoV-2) [2,3,4]. Emerging in late 2019, the novel Coronavirus Disease 2019 (COVID-19) caused by SARS-CoV-2 has disseminated rapidly around the globe and has been declared a pandemic [5,6]. By 27 March 2020, 465,915 confirmed cases have been reported to the World Health Organization (WHO) from 200 countries or territories, with 21,031 deaths (https://www.who.int/emergencies/diseases/novel-coronavirus-2019). The case fatality rate estimate ranged from about 1% in resource-rich settings, to up to 12% in epicentres [6]. Multiple clinical trials evaluating known and novel pharmacological agents have been in progress [7,8,9,10], and innovative epidemiological modelling and mechanistic exploratory studies have also been made available [11,12,13,14]; at the time of writing, there is no effective antiviral therapy of proven clinical benefit.

While widespread global transmission seemed inevitable, most countries continued to implement a containment strategy as advised by the WHO [15,16]; to this end, early testing and diagnosing symptomatic and asymptomatic infectious individuals [4,17], as well as testing of apparently recovered patients who may continue to shed the virus via various routes [18,19,20] remains essential to outbreak control. Various groups have made publicly available broadly-specific assays that target pan-coronaviruses, various members of the *Sarbecovirus* subgenus, or modified assays with specific oligonucleotide probes to achieve SARS-CoV-2 discrimination (https://www.who.int/emergencies/diseases/novel-coronavirus-2019/technical-guidance/laboratory-guidance). With the identification of SARS-CoV-2 as the culprit viral species, a highly-specific assay, without cross-reactivity to closely-related viruses infecting humans such as the severe acute respiratory syndrome coronavirus (SARS-CoV) [21] and Middle East respiratory syndrome coronavirus (MERS-CoV) [22], is needed.

In this study, using the in-house developed program GolayMetaMiner (https://github.com/hkhcc/GolayMetaMiner) and our previously obtained SARS-CoV-2 genome data [23], we attempted to deduce species-specific targets that are conserved among globally detected SARS-CoV-2 isolates. With the identified targets, primers were designed and optimized for a highly sensitive real-time reverse transcription-polymerase chain reaction (RT-PCR) assay, eventually without the additional use of fluorescent reporter probes. The optimized assay was then evaluated on a variety of patient specimens. Finally, the potential application and significance of the newly-developed assay, as well as the known limitations, were discussed.

## 2. Results

### 2.1. Species-Specific SARS-CoV-2 Genomic Regions Identified by GolayMetaMiner

With default settings on an Intel^®^ Core™ i7-2600 desktop computer equipped with a solid-state hard disk drive (k-mer size = 12, number of threads = 8), the genome subtraction run completes in about 25 s, after initial genome download from NCBI. As default parameters for bacterial target identification (uniqueness percentile cutoff = 99.99th centile) failed to report any targets with length > 50 nt, the uniqueness percentile score cutoff was progressively relaxed to the 98th centile considering the genome length of HKU-SZ-005b (29,891 bp).

**Figure 1:**
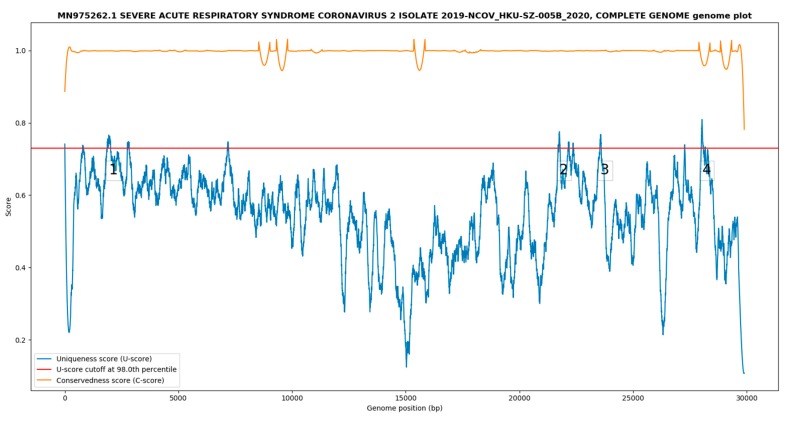
Genome uniqueness/conservedness plot generated by GolayMetaMiner. The orange curve denotes the Savitzky–Golay smoothed conservedness score (C-score), and the blue curve and red line denote the similarly smoothed uniqueness score (U-score) with the corresponding cutoff in the 98th percentile cutoff score. Areas of the blue curve above the cutoff represent the severe acute respiratory syndrome coronavirus 2 (SARS-CoV-2) unique targets, potentially. The genomic targets (> 50 nt) identified were marked by the numbers 1, 2, 3 and 4 in the figure as part of the program output.

The genome uniqueness/conservedness plot is shown in Figure 1. A total of four SARS-CoV-2 unique targets >50 nt in length (Table 1) were reported while other potential targets were too short and excluded by the program. The minimum, median and maximum uniqueness scores (U-scores) were 0.106, 0.566 and 0.810 with a 98th percentile cutoff at 0.730. The minimum, median and maximum conservedness scores (C-scores) were 0.782, 0.999 and 1.031 (> 1 due to overshoot of the 3rd order polynomial in the smoothing function).

### 2.2. Primer Selection for the SARS-CoV-2-Specific Real-Time RT-PCR Assay

The four reported targets (Table 1) were subjected to analysis using the GenScript real-time PCR (TaqMan) Primer and Probes Design Tool (https://www.genscript.com/tools/real-time-pcr-taqman-primer-design-tool) with default settings. Only one of the targets fulfilled the primer and probe design criteria (Target 1, Table 1). After initially optimizing the hydrolysis probe-based version of the assay to show that it reached the sensitivity of our previously published COVID-19-RdRp/Hel assay (data not shown), we used only the primers (nsp2f: 5′-ATGCATTTGCATCAGAGGCT-3′, nsp2r: 5′-TTGTTATAGCGGCCTTCTGT-3′), to develop the subsequent probe-free, COVID-19-nsp2 real-time RT-PCR assay.

### 2.3. Analytical Sensitivity of the Novel COVID-19-nsp2 Real-Time RT-PCR Assay

To determine the analytical sensitivity of the COVID-19-nsp2 assay, the limit of detection (LOD) was evaluated by using viral genomic RNA extracted from a culture isolate of SARS-CoV-2. Serial 10-fold dilutions of SARS-CoV-2 RNA extracted from the viral isolate were prepared and tested in triplicate in two independent runs. The LOD of the COVID-19-nsp2 assay was 1.8 TCID_50_/mL (Table 2).

### 2.4. Analytical Specificity of the COVID-19-nsp2 Assay

To investigate whether the novel COVID-19-nsp2 assay would non-specifically amplify other human-pathogenic coronaviruses and respiratory viruses, we tested total nucleic acid (TNA) extracted from a clinical respiratory specimen with HCoV-HKU1, and TNAs extracted from 17 culture isolates of SARS-CoV, MERS-CoV, HCoV-OC43, HCoV-229E, HCoV-NL63, respiratory syncytial virus, human metapneumovirus, influenza A ((H1N1)pdm09 and H3N2) viruses, influenza B virus, influenza C virus, parainfluenza viruses types 1 to 4, rhinovirus and human adenovirus. This assay did not show cross reactivity with these viruses.

### 2.5. Imprecision of the COVID-19-nsp2 Assay

Using TNA extracted from SARS-CoV-2 isolate at different concentrations, the new COVID-19-nsp2 assay was performed in triplicate for each concentration to evaluate the intra- and inter-assay variations. The total imprecision (% CV) was 1.19% at the lowest concentration tested (Table 3).

### 2.6. Diagnostic Performance Evaluation of the COVID-19-nsp2 Assay for the Detection of SARS-CoV-2 RNA in Clinical Specimens

To evaluate the diagnostic performance of the assay, 59 clinical specimens (23 positive and 36 negative) from 14 confirmed cases (defined as at least one respiratory specimen positive for SARS-CoV-2 by our previously established COVID-19-RdRp/Hel assay) [24] were used. The negative specimens included extrapulmonary (e.g., urine, rectal swab) and respiratory tract specimens that were collected during the later phase of illness when the viral load gradually decreased/became negative. Twenty-three were positive (including 22 respiratory specimens and 1 stool specimen, with Cp values ranged from 18.69 to 36.21) and 36 were negative (including four respiratory and 32 non-respiratory specimens) by the COVID-19-nsp2 assay. The melting curve from COVID-19-nsp2-positive specimens showed a unique peak with the melting temperature of around 80 °C. The results from the two assays were 100% concordant, and no additional positive result was detected from the specimens previously tested negative by the COVID-19-RdRp/Hel assay.

## 3. Discussion

Early in the COVID-19 outbreak, when the virus was reported to be related to the SARS-CoV and the exact genomic variation of the virus was not adequately known [25], multiple groups developed and published assays based on conserved regions that could potentially detect additional coronaviruses [26,27] (see also https://www.who.int/emergencies/diseases/novel-coronavirus-2019/technical-guidance/laboratory-guidance). The advantage of the traditional approach was two-fold: first, it was a more certain method to detect a novel member of the clade and avoided missing cases clinically; second, as SARS-CoV-2 positive control specimens were not globally available at the beginning of the pandemic, laboratories that did not have a species-specific positive control could use other existing positive controls such as SARS-CoV to ensure the performance of their assays.

In the present study, we demonstrated that the diagnostic performance of the hydrolysis probe-free COVID-19-nsp2 assay was comparable to that of our previously developed COVID-19-RdRp/Hel assay [24]. A major novelty of the current approach was the application of genome subtraction to deduce a previously untargeted region of the viral genome encoding nsp2 and subsequent adoption in a diagnostic assay. To our knowledge, no SARS-CoV-2 assay targeting the nsp2 coding region has been published previously. The current nsp2 assay was highly specific (without cross-reaction with other common respiratory viruses) and comparable to the COVID-19-RdRp/Hel assay in terms of analytical sensitivity. The diagnostic sensitivity and specificity of the nsp2 assay was 100% in comparison with the COVID-19-RdRp/Hel assay in this study. The reproducibility, in terms of Cp values, was satisfactory with both intra- and inter-assay coefficient of variation values well below the 5% cutoff specified in literature [28,29]. Furthermore, the PCR reaction time of the nsp2 assay was within an hour, which is shorter than that of the previously developed COVID-19-RdRp/Hel assay [24]; this is a distinct advantage in clinical laboratories, as rapid results can facilitate the timely triage of suspected COVID-19 cases and guide infection control and patient management. In terms of functional significance, the nsp2 region highlighted by genome subtraction corroborated with a recent modeling study which suggested that positively selected regions within the nsp2 protein could contribute to the pathogenicity of the virus [30].

As COVID-19 unfolded into a pandemic, rapid, robust and sensitive diagnostic testing became a priority in containment-based control strategies. While antibody-based testing has been developed [31] and even made commercially available as point-of-care testing kits [32], they lack sensitivity in detecting early infection before the host has successfully mounted a humoral response [33], cannot be applied to certain specimen types (such as urine [19], as antibodies cannot be secreted into the glomerular filtrate unless the patient develops severe renal damage) and cannot be used to determine when the host ceases to become infectious [34]. As nucleic acid amplification-based testing allows exquisite sensitivity (1.8 TCID_50_/mL in our case) and can be readily adopted in laboratories with basic molecular facilities, it is an essential tool in the clinical management and triage of patients. The robust design of the optimized assay, requiring only one pair of SARS-CoV-2-specific primers to diagnose COVID-19 infection in humans, avoids the potential logistics, reagent and manpower constraints in performing multiple or cascade testing or the use of relatively costly reporter probes. In developed countries where medical resources have become critically limited [35,36], it is believed that this new assay may be adopted to allow for the testing of more patients in a shorter time.

While we have validated the primer set against all publicly available SARS-CoV-2 genomes (Supplementary Materials) and found the proposed nsp2 target to be 100% conserved, it is possible that with random genetic drift, certain sub-clones of the virus may eventually develop mutations and escape detection. As SARS-CoV-2 is an RNA virus, which typically has a relatively high mutation rate (10^−4^ nucleotide substitutions per site per year for coronaviruses [37]), this is a distinct possibility that must be considered. However, with the use of less-specific assays, such as pan-coronavirus primers [38], the diagnostic laboratory can adopt a two-tier approach by first attempting to confirm infection using the new nsp2 assay, and subsequently use the broadly-specific assays on clinically suspicious cases to rule out SARS-CoV-2 infection. Finally, GolayMetaMiner is available as a free and open-source software that can be applied efficiently to newly deduced mutant sequences. Using the software tool, degenerate bases may be strategically incorporated into the COVID-19-nsp2 assay primers to broaden the specificity, or additional targets may be iteratively identified from further coronavirus pan-genome analyses.

## 4. Materials and Methods

### 4.1. SARS-CoV-2 Genome Subtraction Using GolayMetaMiner

The GolayMetaMiner software was initially developed with the School of Biomedical Sciences, The Chinese University of Hong Kong as an in-house software for the design of species- and species-group specific molecular assays for *Mycobacterium* species for the Department of Microbiology, Queen Mary Hospital, and was improved upon the a previously published software, ssGeneFinder [39,40,41]. The GolayMetaMiner software first downloads the genomes of the target and non-target species from the NCBI nucleotide database and generates a pool of nucleotide k-mers (k = 12 by default) for both forward and reverse directions of the non-target genomes. Next, the program steps through the forward strand of the target genome (--primary_target) and assign a binary uniqueness value of either 1 (k-mer is unique to the target genome) or 0 (k-mer is found in the non-target pool). As a pathogen detection assay needs to account for potential genomic variability of the target pathogen, the GolayMetaMiner software then steps through the target genome to determine if the k-mer is also present in other intended targets (--secondary_targets or --secondary_target_list), adding a value of 1 (k-mer is present in a secondary target genome) or 0 (k-mer is absent in a secondary genome). The conservedness value is normalized to a maximum value of 1 by dividing this absent/present count by the total number of secondary target genomes. Before plotting the scores, the arrays of uniqueness and conservedness values are smoothed using an empirically optimized Savitzky–Golay filter (window size = 501, polynomial order = 3) and transformed to continuous scores (U-score, C-score), and a U-score cutoff is calculated according to a specified percentile cutoff (defaults to 99.99th).

In this study, GolayMetaMiner was executed under an Anaconda Python 3.7 environment (freely available from https://www.anaconda.com/distribution/) with the command line as follows: python gmm.py --primary_target MN975262.1 --secondary_target_list SARS-CoV-2_genomes.txt--non_target_ncbi_table coronaviridae_complete.csv --exclusion_string “Severe acute respiratory syndrome coronavirus 2” --reporting_centile 98. The longer of the two SARS-CoV-2 genomes sequenced by our group [23], HKU-SZ-005b, was used as the reference genome (--primary_target MN975262), and a list of accession numbers of publicly available SARS-CoV-2 genomes were used as secondary targets (--secondary_target_list SARS-CoV-2_genomes.txt); the accession number of the reference genome was manually commented out with a “#” to avoid double-counting the reference genome in estimating conservedness. Non-targets were specified by downloading a table of complete *Coronaviridae* genomes from the NCBI genome browser (https://www.ncbi.nlm.nih.gov/genome/browse#!/viruses/) and passed to the program as a CSV file (--non_target_ncbi_table coronaviridae_complete.csv). An exclusion string was added to exclude entries marked as SARS-CoV-2 from the genome table (--exclusion_string “Severe acute respiratory syndrome coronavirus 2”). The genome U-score cutoff was empirically determined: if the default setting of 99.99th percentile could not yield any potential target, it would be progressively relaxed to 99.9th, 99th, 98th etc. As genome data could be automatically downloaded when the GolayMetaMiner was executed, no genome data was included with the software distribution to respect the rights of certain sequence owners. The lists of accession numbers of the 96 SARS-CoV-2 and 104 non-SARS-CoV-2 coronavirus genomes are available in Supplementary Materials. The complete Python 3 source code of the GolayMetaMiner software is freely available from the GitHub repository https://github.com/hkhcc/GolayMetaMiner.

### 4.2. Viruses and Clinical Specimens

SARS-CoV-2 was isolated from the nasopharyngeal aspirate specimen of a laboratory-confirmed COVID-19 patient in Hong Kong as previously described [42]. The viral isolate stock (1.8 × 10^7^ 50% tissue culture infective doses [TCID_50_]/mL) was prepared using VeroE6 cells as previously described [20,24,43]. For analytical sensitivity evaluation, TNA extracted from SARS-CoV-2 isolate was used. For analytical specificity evaluation, a clinical respiratory specimen of HCoV-HKU1 and 17 culture isolates of other human-pathogenic coronaviruses and respiratory viruses were used [24]. For assay performance evaluation, 59 archived clinical specimens (26 respiratory specimens including nasopharyngeal aspirate/swab, throat swab, endotracheal aspirate, sputum and saliva, and 33 non-respiratory specimens including plasma, urine, rectal swab/stool) from 14 patients with laboratory-confirmed COVID-19 were used [24]. These specimens were evaluated previously using the established COVID-19-RdRp/Hel assay [24]. The study was approved by Institutional Review Board of The University of Hong Kong/Hospital Authority (UW 13-372).

### 4.3. Nucleic Acid Extraction and RT-PCR for SARS-CoV-2

TNA extraction from clinical specimens and viral culture isolates was performed using NucliSENS easyMAG extraction system (BioMerieux, Marcy-l’Étoile, France) [24,44]. The volume of the specimens used for extraction and the elution volume depended on the specimen type and the amount of the specimen available as previously described [28,45,46].

Real-time RT-PCR assay for SARS-CoV-2 RNA detection was performed using QuantiNova SYBR Green RT-PCR Kit (QIAGEN, Hilden, Germany) and a LightCycler 480 II real-time PCR System (Roche, Basel, Switzerland) [4,20]. Each 20 μL reaction mixture contained 10 μL of 2× QuantiNova SYBR Green RT-PCR Master Mix, 0.2 μL of QN SYBR Green RT-Mix, 1 μL of each 10 μM forward and reverse primer, 2.8 μL of RNase-free water and 5 μL of TNA as the template. The thermal cycling condition was 10 min at 50 °C and 2 min at 95 °C, followed by 45 cycles of 5 s at 95 °C and 10 s at 60 °C, and then subjected to melting curve analysis (95 °C for 5 s, 65 °C for 1 min, followed by a gradual increase in temperature to 97 °C with continuous recording of fluorescence).

## 5. Conclusions

Using GolayMetaMiner genome subtraction, SARS-CoV-2-specific regions were successfully identified using 96 SARS-CoV-2 and 104 non-SARS-CoV-2 coronavirus genomes. Identified regions included a 154-nt conserved sequence in the nsp2 gene, which was absent in other human-pathogenic coronaviruses and has not previously been targeted in real-time RT-PCR assays of COVID-19. The highly specific and sensitive nsp2-based assay was validated using multiple viral culture isolates and clinical specimens. The newly developed assay, COVID-19-nsp2, and the associated genomic findings in this study will contribute to the control and understanding of the current COVID-19 outbreak.

## Figures and Tables

**Table 1:** SARS-CoV-2 specific targets (>50 nt) reported by GolayMetaMiner.

Target	Nucleotide Position ^1^	Target Length (nt)	Genomic Region
1	1865–2018	154	nsp2
2	21,731–21,788	58	Spike
3	23,536–23,598	63	Spike
4	27,997–28,909	93	ORF8

^1^ With reference to SARS-CoV-2 isolate HKU-SZ-005b_2020, accession MN975262.1.

**Table 2:** Evaluation of the limit of detection (LOD) of the COVID-19-nsp2 real-time reverse transcription-polymerase chain reaction (RT-PCR) assay using SARS-CoV-2 genomic RNA from cell culture lysate.

Virus Quantity (TCID_50_/mL)	Cp (Intra-Run)			Cp (Inter-Run)		
	Test 1	Test 2	Test 3	Test 1	Test 2	Test 3
1.8 × 10^2^	29.91	30.12	29.90	29.23	29.54	29.28
1.8 × 10^1^	33.55	33.49	33.78	32.41	32.95	32.69
1.8 × 10^0^	37.39	37.31	37.20	36.72	36.25	37.20
1.8 × 10^−1^	-	-	-	-	38.96	-

Cp: crossing point at which the fluorescence of a sample rises above the background fluorescence.

**Table 3:** Imprecision testing of the COVID-19-nsp2 assay using SARS-CoV-2 isolate extracts.

Virus Quantity (TCID_50_/mL)	Intra-Assay		Inter-Assay
	No. of Positive Replicates	Mean Cp ± SD (% Coefficient of Variation)	Mean Cp ± SD (% Coefficient of Variation)
1.8 × 10^2^	3	29.98 ± 0.12 (0.41)	29.66 ± 0.37 (1.24)
1.8 × 10^1^	3	33.61 ± 0.15 (0.46)	33.15 ± 0.54 (1.64)
1.8 × 10^0^	3	37.30 ± 0.10 (0.26)	37.01 ± 0.44 (1.19)

## Supplementary Materials

Supplementary materials can be found at https://www.mdpi.com/1422-0067/21/7/2574/s1. Supplementary File 1. List of NCBI GenBank accession numbers for target and non-target genome used for analysis.

## Abbreviations

COVID-19	Novel coronavirus infection
SARS-CoV-2	Severe acute respiratory syndrome coronavirus 2
WHO	World Health Organization
HCoV	Human coronavirus
SARS-CoV	Severe acute respiratory syndrome coronavirus
MERS-CoV	Middle East respiratory syndrome coronavirus
RT-PCR	Reverse transcription-polymerase chain reaction
NCBI	National Center for Biotechnology Information, USA
U-score(s)	Uniqueness score(s)
C-score(s)	Conservedness score(s)
LOD	Limit of detection
TNAnt	Total nucleic acidnucleotide
