# Supplementary material for: Development of a Novel, Genome Subtraction-Derived, SARS-CoV-2-Specific COVID-19-nsp2 Real-Time RT-PCR Assay and Its Evaluation Using Clinical Specimens
Source: Int J Mol Sci. 2020 Apr 8;21(7):2574. doi: 10.3390/ijms21072574 (PMC7177594; doi:10.3390/ijms21072574)
Supplement: Supplementary file 1 [file ijms-21-02574-s001.pdf]

Supplementary File 1

List of NCBI GenBank accession numbers for target and non-target genome used for analysis

1. **Target genomes (SARS-CoV-2)**
   The primer target (MN975262.1) was marked with a "#" to exclude from conservedness score calculation.

```
MT126808.1
MT007544.1
MT192773.1
MT192772.1
MT192765.1
MN996531.1
MN996530.1
MN996529.1
MN996528.1
MN996527.1
MN908947.3
MT192759.1
MT188341.1
MT188340.1
MT188339.1
MT123293.2
MT123292.2
MT123291.2
MT093631.2
NC_045512.2
MT184913.1
MT184912.1
MT184911.1
MT184910.1
MT184909.1
MT184908.1
MT184907.1
LC529905.1
MT163719.1
MT163718.1
MT163717.1
MT163716.1
MT159722.1
MT159721.1
MT159720.1
MT159719.1
MT159718.1
MT159717.1
MT159716.1
MT159715.1
MT159714.1
MT159713.1
MT159712.1
MT159711.1
```

MT159710.1
MT159709.1
MT159708.1
MT159707.1
MT159706.1
MT159705.1
MT121215.1
MT066156.1
MT050493.1
MT012098.1
MT152824.1
MT135044.1
MT135043.1
MT135042.1
MT135041.1
LC528233.1
LC528232.1
MT123290.1
MT118835.1
MT106054.1
MT106053.1
MT106052.1
MT093571.1
MT072688.1
MT066176.1
MT066175.1
MT049951.1
MT044258.1
MT044257.1
MT039888.1
MT039887.1
MT039890.1
MT039873.1
MT027064.1
MT027063.1
MT027062.1
MT020881.1
MT020880.1
MT019533.1
MT019532.1
MT019531.1
MT019530.1
MT019529.1
MN997409.1
MN994468.1
MN994467.1
MN988713.1
MN988669.1
MN988668.1
MN985325.1
MN975262.1

MN938384.1

2. **Non-target genomes (coronaviruses other than SARS-CoV-2)**
   The SARS-CoV-2 genomes were excluded by the program using the switch --exclusion_string "Severe acute respiratory syndrome coronavirus 2".

Organism Name	Replicons
Bat coronavirus BM48-31/BGR/2008	NC_014470.1/GU190215.1
Sparrow coronavirus HKU17	NC_016992.1/JQ065045.1
Porcine coronavirus HKU15	NC_039208.1/JQ065043.2
White-eye coronavirus HKU16	NC_016991.1/JQ065044.1
Magpie-robin coronavirus HKU18	NC_016993.1/JQ065046.1
Night heron coronavirus HKU19	NC_016994.1/JQ065047.1
Wigeon coronavirus HKU20	NC_016995.1/JQ065048.1
Common moorhen coronavirus HKU21	NC_016996.1/JQ065049.1
Thrush coronavirus HKU12-600	NC_011549.1/FJ376621.1
Munia coronavirus HKU13-3514	NC_011550.1/FJ376622.1
Ferret coronavirus	LC119077.1
Bat coronavirus	NC_034440.1/KX574227.1
Bulbul coronavirus HKU11-934	NC_011547.1/FJ376619.2
unidentified human coronavirus	MF996621.1
Porcine coronavirus HKU15	JQ065042.2
Human coronavirus 229E	NC_002645.1/AF304460.1
Porcine epidemic diarrhea virus	NC_003436.1/AF353511.1
Human coronavirus NL63	NC_005831.2/AY567487.2
Human coronavirus HKU1	NC_006577.2/AY597011.2
Scotophilus bat coronavirus 512	NC_009657.1/DQ648858.1
Rhinolophus bat coronavirus HKU2	NC_009988.1/EF203064.1
Miniopterus bat coronavirus HKU8	NC_010438.1/EU420139.1
Beluga whale coronavirus SW1	NC_010646.1/EU111742.1
Bat coronavirus HKU4-1	NC_009019.1/EF065505.1
Bat coronavirus HKU5-1	NC_009020.1/EF065509.1
Bat coronavirus HKU9-1	NC_009021.1/EF065513.1
Murine hepatitis virus	AY700211.1
Human coronavirus OC43	NC_006213.1/AY585228.1
Rabbit coronavirus HKU14	NC_017083.1/JN874559.1
Rousettus bat coronavirus HKU10	NC_018871.1/JQ989270.1
Bat coronavirus CDPHE15/USA/2006	NC_022103.1/KF430219.1
Betacoronavirus Erinaceus/VMC/DEU/2012	NC_039207.1/KC545383.1
Bat Hp-betacoronavirus/Zhejiang2013	NC_025217.1/KF636752.1

Betacoronavirus HKU24	NC_026011.1/KM349742.1
Swine enteric coronavirus	NC_028806.1/KR061459.1
BtRf-AlphaCoV/YN2012	NC_028824.1/KJ473808.1
BtRf-AlphaCoV/HuB2013	NC_028814.1/KJ473807.1
BtNv-AlphaCoV/SC2013	NC_028833.1/KJ473809.1
BtMr-AlphaCoV/SAX2011	NC_028811.1/KJ473806.1
Rousettus bat coronavirus	NC_030886.1/KU762338.1
Mink coronavirus strain WD1127	NC_023760.1/HM245925.1
NL63-related bat coronavirus	KY073745.1
Lucheng Rn rat coronavirus	NC_032730.1/KF294380.2
Coronavirus AcCoV-JC34	NC_034972.1/KX964649.1
Wencheng Sm shrew coronavirus	NC_035191.1/KY967717.1
Miniopterus bat coronavirus 1	NC_010437.1/EU420138.1
NL63-related bat coronavirus	NC_032107.1/KY073744.1
Ferret coronavirus	NC_030292.1/KM347965.1
Swine enteric coronavirus	LT545990.1
Betacoronavirus Erinaceus/VMC/DEU/2012	KC545386.1
Turkey coronavirus	NC_010800.1/EU095850.1
Murine hepatitis virus	NC_001846.1/AF029248.1
Porcine epidemic diarrhea virus	LT897799.1
Porcine epidemic diarrhea virus	LT900501.1
Rat coronavirus Parker	NC_012936.1/FJ938068.1
Porcine epidemic diarrhea virus	LT898427.1
Porcine epidemic diarrhea virus	LT898426.1
Porcine epidemic diarrhea virus	LT898438.1
Porcine epidemic diarrhea virus	LT898440.1
Porcine epidemic diarrhea virus	LT898420.1
Porcine epidemic diarrhea virus	LT898409.1
Porcine epidemic diarrhea virus	LT900498.1
Porcine epidemic diarrhea virus	LT898441.1
Porcine epidemic diarrhea virus	LT898430.1
Porcine epidemic diarrhea virus	LT898446.1
Porcine epidemic diarrhea virus	LT898411.1
Porcine epidemic diarrhea virus	LT898413.1
Porcine epidemic diarrhea virus	LT898436.1
Porcine epidemic diarrhea virus	LT898431.1
Porcine epidemic diarrhea virus	LT898444.1
Porcine epidemic diarrhea virus	LT898410.1
Porcine epidemic diarrhea virus	LT898423.1
Porcine epidemic diarrhea virus	LT898412.1
Porcine epidemic diarrhea virus	LT900500.1

Porcine epidemic diarrhea virus	LT898415.1
Porcine epidemic diarrhea virus	LT898417.1
Porcine epidemic diarrhea virus	LT898421.1
Porcine epidemic diarrhea virus	LT900502.1
Porcine epidemic diarrhea virus	LT898416.1
Porcine epidemic diarrhea virus	LT898435.1
Porcine epidemic diarrhea virus	LT898445.1
Porcine epidemic diarrhea virus	LT898439.1
Porcine epidemic diarrhea virus	LT898414.1
Porcine epidemic diarrhea virus	LT900499.1
Porcine epidemic diarrhea virus	LT898425.1
Porcine epidemic diarrhea virus	LT898432.1
Porcine epidemic diarrhea virus	LT898408.1
Porcine epidemic diarrhea virus	LT898418.1
Porcine epidemic diarrhea virus	LT898443.1
Porcine epidemic diarrhea virus	LT898433.1
Porcine epidemic diarrhea virus	LT898447.1
Porcine epidemic diarrhea virus	LT906582.1
Porcine epidemic diarrhea virus	LT906620.1
Porcine epidemic diarrhea virus	LT905450.1
Porcine epidemic diarrhea virus	LT905451.1
Transmissible gastroenteritis virus	NC_038861.1/AJ271965.2
Infectious bronchitis virus	NC_001451.1/M95169.1
Severe acute respiratory syndrome-related coronavirus	NC_004718.3/AY274119.3
Human betacoronavirus 2c EMC/2012	NC_019843.3/JX869059.2
Betacoronavirus England 1	NC_038294.1/KC164505.2
Camel alphacoronavirus	NC_028752.1/KT368907.1
Severe acute respiratory syndrome coronavirus 2	MN938384.1
Bovine coronavirus	NC_003045.1/AF391541.1
Feline infectious peritonitis virus	NC_002306.3/AY994055.1
Severe acute respiratory syndrome coronavirus 2	MN975262.1
Severe acute respiratory syndrome coronavirus 2	MN985325.1
Severe acute respiratory syndrome coronavirus 2	MN988713.1
Severe acute respiratory syndrome coronavirus 2	MN994467.1
Severe acute respiratory syndrome coronavirus 2	MN994468.1

Severe acute respiratory syndrome coronavirus 2	MN997409.1
Severe acute respiratory syndrome coronavirus 2	MN988668.1
Severe acute respiratory syndrome coronavirus 2	MN988669.1
Severe acute respiratory syndrome coronavirus 2	MN996527.1
Severe acute respiratory syndrome coronavirus 2	MN996528.1
Severe acute respiratory syndrome coronavirus 2	MN996529.1
Severe acute respiratory syndrome coronavirus 2	MN996530.1
Severe acute respiratory syndrome coronavirus 2	MN996531.1
Severe acute respiratory syndrome coronavirus 2	NC_045512.2/MN908947.3
